# Modified prone position to dissect the popliteal fossa

**DOI:** 10.1308/003588413X13511609955779

**Published:** 2013-03

**Authors:** A Boulemden, T Ritzmann, S Liptrot, A Abbas, KR Makhdoomi

**Affiliations:** Sherwood Forest Hospitals NHS Foundation Trust, UK

**Keywords:** Modified prone position, Popliteal fossa dissection, Airway

## Abstract

**Introduction:**

Traditionally, the prone position is used for dissecting the popliteal fossa, which requires endotracheal intubation. Access to the airway in this position is limited, hence the complications. It is not surprising that the prone position is not favoured by the anaesthetists, especially in patients with a high body mass index. We describe a safe and novel alternative to the prone position.

**Methods:**

The modified prone position (MPP) is described as an alternative position that facilitates access to the airway.

**Results:**

Between October 2007 and May 2010, 12 patients underwent popliteal fossa dissection using the MPP. All patients had general anaesthesia using a laryngeal mask airway with the exception of one, who had an epidural anaesthesia. There were no airway or haemodynamic complications. The surgical access to the popliteal fossa was as good as with the traditional prone position.

**Conclusions:**

The MPP was satisfactory for both the surgeon and the anaesthetists. The authors now use this position routinely for dissecting the popliteal fossa.

Traditionally, the prone position has been used to dissect the popliteal fossa, although this position is associated with a number of physiological changes with a high risk of complications.^1^ Wadsworth *et al* published the effects of different surgical prone positions on cardiovascular parameters.^1^ They reported a 20% decrease in the cardiac index of the patients in the knee-chest position and a 17% decrease in the props position.

Obstruction of the inferior vena cava is another well recognised problem, leading to reduced cardiac output, increased bleeding risk, venous stasis and, consequently, thrombotic complications.^2^ In addition, blindness has also been reported as a complication of the prone position.

The most serious complication of the prone position is the possibility of airway loss in anaesthetised patients, which can be life threatening.^3^ Accidental endotracheal extubation has also been reported.^4^

## Methods

A review of a prospectively gathered database of all patients who underwent exploration of the popliteal fossa in a modified prone position (MPP) in our hospital was performed to assess the safety and practicability of this position. The Embase™, MEDLINE^®^ and the Cochrane Library databases were searched until the end of July 2010 by entering the phrases ‘popliteal artery*’, ‘popliteal fossa*’, ‘patient position*’ and ‘prone position*’. The position described in this paper has not been reported in the vascular literature. Student’s t-test was used for statistical analysis.

**Figure 1:**
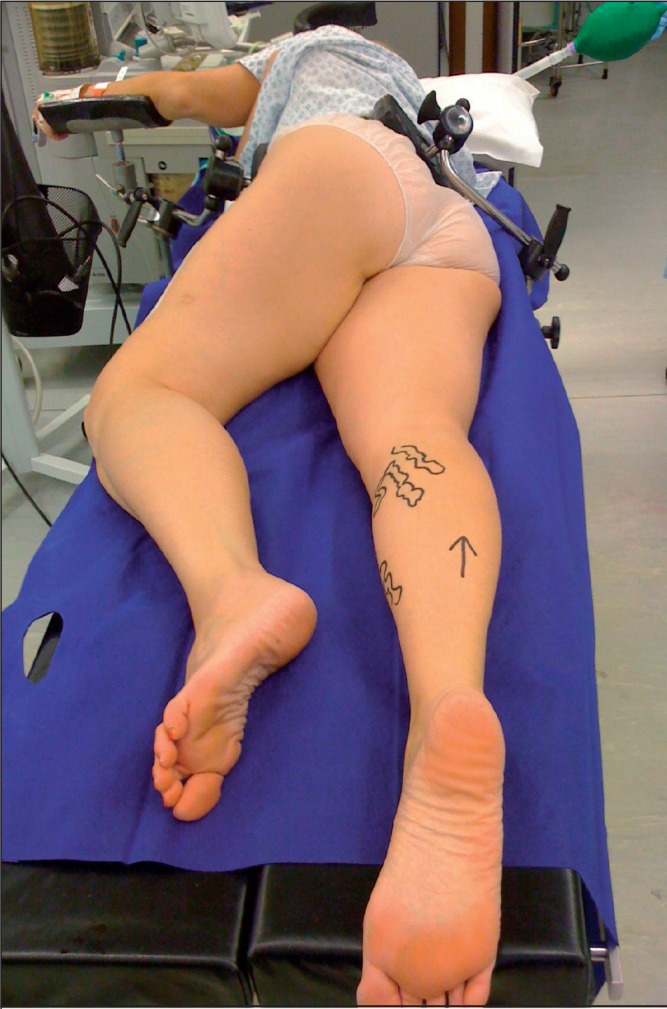
The modified prone position

The MPP consists of placing the patient in the lateral position, facing the side on which the operation is to be performed. The patient’s head, neck and upper thoracic spine are maintained in this nearly lateral position, with the arm being supported by an arm board attached to the operating table. Rotating the patient’s lower thoracic spine, lumbar spine and hips through an angle approaching 45º brings the popliteal fossa into a prone position. In addition to stabilisation provided by the arm board, an attachment is used to stabilise the patient’s hips and a strap is applied around the waist. Gel pads are sited at points where the patient is in contact with the table or an attachment (Fig 1).

## Results

Between October 2007 and May 2010, 12 patients (6 men, 6 women; mean age: 57 years) underwent dissection of the popliteal fossa in the MPP. Ten operations were performed for saphenopopliteal junction ligation and two for popliteal aneurysms. All procedures were performed by the same surgeon and anaesthetist. All patients had the operations under general anaesthesia using a laryngeal mask airway (LMA^®^; Intavent Direct, Maidenhead, UK) with the exception of one, who had the operation under epidural anaesthesia. Patients were followed up at three months after the operation. There were no immediate complications. One patient developed pneumonia three weeks after the operation and was treated with intravenous antibiotics, with good recovery. Access to the airway was not an issue and there were no airway complications in any of the patients. There were no concerns about access to the popliteal fossa and no technical difficulties reported by the surgeon.

All patients had their systolic blood pressure, pulse, oxygen saturation and end-tidal CO_2_ measured before and after being placed in the MPP. There was no significant difference between the measurements before and after (Table 2). In the supine position, the mean systolic blood pressure was 116mmHg (range: 78–150mmHg, standard deviation [SD]: 17mmHg), the mean oxygen saturation was 99% (range: 98–100%, SD: 0.51%) and the mean pulse was 68bpm (range: 58–72bpm, SD: 5bpm). In the MPP, the mean systolic blood pressure was 114mmHg (range: 92–148mmHg, SD: 14mmHg), the mean oxygen saturation was 99% (range: 97–100%, SD: 1%) and the mean pulse was 69bpm (range: 61–75bpm, SD: 5bpm).

**Table 1:** Different complications related to popliteal fossa surgery in the prone position

**Cardiovascular**	Inferior vena cava obstruction Venous thromboembolism Compression of peripheral vessels (axillary artery) Limb compartment syndrome and rhabdomyolysis
**Abdominal**	Visceral ischaemia in prolonged prone position
**Nervous system**	Peripheral nerve injuries Cervical spine injuries
**Pressure injuries**	Tracheal compression Macroglossia and oropharyngeal swelling Contact dermatitis of the face
**Musculoskeletal**	Shoulder dislocation

**Table 2:** Haemodynamic and respiratory parameters with supine and modified prone position (*n*=12)

Parameters	Supine position	Modified prone position	*P*-value
Mean systolic blood pressure	116mmHg	114mmHg	0.711
Mean pulse	68bpm	69bpm	0.568
Mean oxygen saturation	99%	99%	0.754
Mean end-tidal CO_2_ pressure (*n*=7)	5.8kPa	6.6kPa	0.969

## Discussion

Surgery of the popliteal fossa is traditionally performed in the prone position. Owing to the inaccessibility of the airway and potential life threatening complications, this position is not favoured by anaesthetists, who ask: ‘Can this operation be performed in the lateral position?’ This reluctance by the anaesthetists to place the patient in the prone position is not unfounded.

It is well recognised that inferior vena cava pressure is raised in the prone position.^5^ Edgcombe *et al* discussed the effects of inferior vena cava obstructions in the prone position.^2^ They found an increased risk of reduced cardiac output, bleeding, venous stasis and thrombotic complications. Wadsworth *et al* measured cardiovascular parameters in different prone positions in 20 healthy volunteers and found that the cardiac index was reduced by up to 17–20% compared with these parameters in the supine position.^1^

The major concern of the traditional prone position is the possibility of the loss of airway in an anaesthetised patient with life-threatening complications. Accidental endotracheal extubation has been reported and managed in a number of ways including turning patients back to the supine position and fibreoptic intubation in the prone position.^2^ Many other complications have also been reported in the literature (Table 1).^2^

Keeping this in mind, we wanted to try a different position that could avoid these complications. Initially, it was thought that the access to the popliteal fossa would be compromised with the MPP. However, there was no reported difficulty in accessing the operative field. In the MPP, the upper part of the body is in a lateral position and access to the airway is therefore always maintained. In all patients but one an LMA^®^ was used instead of an endotracheal tube for maintenance of anaesthesia without any tube displacement. In addition, there was no significant change between the systolic blood pressure, pulse, oxygen saturation or endotracheal carbon dioxide levels before and after placing the patient in the MPP (Table 2).

Patients remained stable throughout the procedures with no airway compromise and no haemodynamic complications. However, our study was limited owing to its small numbers, which might explain the non-significant difference in the parameters before and after placing the patient in the MPP.

The MPP can also be achieved under epidural anaesthesia, as with one of our patients. None of our patients had any cardiovascular or respiratory complications and all were discharged home on the same day or a few days after the operation. One patient was readmitted under the medical team with pneumonia three weeks postoperatively.

## Conclusions

This paper describes a novel position to dissect the popliteal fossa that is safe and has no airways or cardiovascular complications. The MPP facilitates simultaneous access to both the popliteal fossa and airway. The authors now routinely use this position for dissecting the popliteal fossa.
